# An open trial of app-assisted acceptance and commitment therapy (*i*ACT) for eating disorders in type 1 diabetes

**DOI:** 10.1186/s40337-020-00357-6

**Published:** 2021-01-06

**Authors:** Rhonda M. Merwin, Ashley A. Moskovich, Michael Babyak, Mark Feinglos, Lisa K. Honeycutt, Jan Mooney, Sara P. Freeman, Heather Batchelder, Devdutta Sangvai

**Affiliations:** 1 Department of Psychiatry and Behavioral Sciences, Duke University Medical Center, DUMC Box 3842, Durham, NC 27712 USA; 2 Department of Medicine, Division of Endocrinology, Metabolism and Nutrition, Duke University Medical Center, Durham, NC USA; 3 Department of Family Medicine and Community Health, Duke University Medical Center, Durham, NC USA

**Keywords:** Eating disorders, Type 1 diabetes, Acceptance and commitment therapy, Experiential avoidance, Psychological flexibility, Diabetes distress, Mobile health

## Abstract

**Background:**

Eating disorders (EDs) among individuals with type 1 diabetes (T1D) increase the risk of early and severe diabetes-related medical complications and premature death. Conventional eating disorder (ED) treatments have been largely ineffective for T1D patients, indicating the need to tailor treatments to this patient population and the unique conditions under which ED symptoms emerge (in the context of a chronic illness with unrelenting demands to control blood glucose, diet and exercise). The current study was a pilot open trial of *i*ACT, a novel intervention for EDs in T1D grounded in Acceptance and Commitment Therapy (ACT). *i*ACT was based on the premise that ED symptoms emerge as individuals attempt to cope with T1D and related emotional distress. *i*ACT taught acceptance and mindfulness as an alternative to maladaptive avoidance and control, and leveraged personal values to increase willingness to engage in T1D management, even when it was upsetting (e.g., after overeating). A tailored mobile application (“app”) was used in between sessions to facilitate the application of ACT skills in the moment that individuals are making decisions about their diabetes management.

**Methods:**

Adults with T1D who met criteria for an ED completed 12 sessions of *i*ACT (with three optional tapering sessions). In addition to examining whether treatment was acceptable and feasible (the primary aim of the study), the study also examined whether *i*ACT was associated with increased psychological flexibility (i.e., the ability to have distressing thoughts/feelings about diabetes while pursuing personally meaningful values), and improvements in ED symptoms, diabetes management and diabetes distress.

**Results:**

Treatment was acceptable to T1D patients with EDs and feasible to implement. Participants reported increased psychological flexibility with diabetes-related thoughts/feelings, and less obstruction and greater progress in pursuing personal values. There were large effects for change in ED symptoms, diabetes self-management and diabetes distress from baseline to end-of-treatment (Cohen’s *d* = .90–1.79). Hemoglobin A_1c_ also improved, but the *p*-value did not reach statistical significance, *p* = .08.

**Conclusions:**

Findings provide preliminary evidence for *i*ACT to improve outcomes for T1D patients with EDs and support further evaluation of this approach in a controlled trial.

**Trial registration:**

NCT02980627. Registered 8 July 2016.

## Plain English summary

Eating disorders (EDs) are fairly common among individuals with type 1 diabetes (T1D) and can be deadly. Treatments for anorexia and bulimia nervosa that are currently available are not as effective for individuals with T1D. This preliminary study examined whether a new treatment, *i*ACT, was acceptable to T1D patients with EDs and feasible to implement, and whether it reduced eating disorder (ED) symptoms and improved diabetes management. *i*ACT was based on Acceptance and Commitment Therapy (ACT) and focused on helping individuals with T1D cope effectively with emotional distress and choose actions based on personal values. A mobile application (“app”) was also used in between sessions to help patients practice new behaviors at home. The treatment was acceptable and feasible, with good participation and few unwanted side effects. Participants reported improvements in their ED symptoms and emotional distress. They also reported improvements in their responses to diabetes-related thoughts and feelings and greater ability to pursue their personal values over the course of treatment, suggesting that treatment worked the way it was intended. The current study suggests that a larger study is needed comparing this treatment to a waitlist (to determine whether improvements are greater than what would happen with time alone) or to other, currently available interventions.

## Background

Type 1 diabetes (T1D) is an endocrine disorder in which the immune system attacks the beta cells of the pancreas, eliminating the body’s ability to produce insulin. Individuals with T1D survive by self-monitoring blood glucose and administering insulin multiple times a day to attempt to achieve near-normal glycemia. It is a complex and burdensome regimen that requires careful planning of diet, exercise and insulin dosing to prevent immediately life-threatening circumstances and slow the progression of macro- and microvascular complications and premature death [1, 2].

Eating disorder (ED) symptomatology is fairly common among adolescents and young women with T1D and has severe negative consequences for diabetes management [3–6]. In a longitudinal study of 126 adolescent girls interviewed 7 times over 14 years, 32.4% (23/71) met the criteria for a current ED at Time 7, and an additional 8.5% (6/71) had a subthreshold ED [4]. The cumulative probability of ED onset was 60% by age 25 [4]. This is consistent with other studies that have found elevated rates of eating disorders (EDs) and disordered eating behavior among young women with T1D (e.g., [5, 6]).

ED symptoms are associated with elevated hemoglobin A_1c_ (HbA_1c_) [6], a key metric of glycemic control that reflects the percentage of hemoglobin in red blood cells that is glycated, i.e. attached to a glucose molecule. HbA_1c_ provides an estimate of average blood glucose over the past 2 to 3 months. Target HbA_1c_ for individuals with T1D is typically between 7.0–7.5% [7]. In previous studies of EDs in T1D mean HbA_1c_ is typically 9% or higher [8]. Higher HbA_1c_ predicts microvascular (e.g., retinopathy, neuropathy, nephropathy) and macrovascular complications (e.g., cardiovascular disease) [2].

ED behaviors in T1D include the dangerous practice of administering less insulin than is needed to maintain or lose weight. While insulin restriction may be effective at producing weight loss (particularly when prolonged), it is extremely dangerous. When insulin is restricted, excess glucose remains in the blood stream, causing hyperglycemia that damages the blood vessels, advancing vascular disease [2]. Glucose is eventually excreted via urination, effectively “purging” calories, but damaging the kidneys. Further, when the body has insufficient insulin, it is unable to use glucose for energy and begins to break down fat for fuel. The breakdown products include ketones that, when released into the bloodstream at high levels, are toxic and can be immediately life-threatening. Food restriction can have a similar, although less severe, effect (with too little glucose available for energy and the body drawing from fat stores, ketones are subsequently released into the bloodstream). When these two behaviors are combined, mortality is particularly high [9]. T1D patients with EDs have an increased incidence of life-threatening diabetic ketoacidosis and hospitalizations [10]. Insulin restriction is the single best predictor of early mortality among individuals with T1D and triples the risk of premature death [11, 12].

Existing treatments for anorexia and bulimia nervosa are largely ineffective for individuals with T1D [13–15]. Even when eating and weight-related attitudes improve, T1D management and glycemic control may not [14]. This suggests the need to tailor interventions to T1D, and the unique needs and experiences of this patient population and the conditions under which ED symptoms emerge.

ED symptomatology is more prevalent among individuals with T1D than the general population [4–6]. Factors that contribute to the increased prevalence of EDs in T1D are unknown, however it is hypothesized that it is directly related to the T1D treatment regimen which encourages perfectionism in blood glucose control achieved via careful carbohydrate counting and intensive insulin therapy [16]. Among vulnerable individuals, attempting to achieve near normal glycemia may lead to maladaptive attention to food choices and attempts to control eating and weight gain associated with insulin therapy [16]. There is empirical evidence that emotional distress specific to diabetes (diabetes distress; e.g., feeling overwhelmed by diabetes or like a failure in diabetes management) could have an important role in EDs in T1D, as both a precipitant and a consequence of disordered eating [12, 17]; however, pathways from this emotional distress to ED symptoms have not been fully specified.

### Hypothesized functional pathways to EDs in T1D

**Fig. 1:**
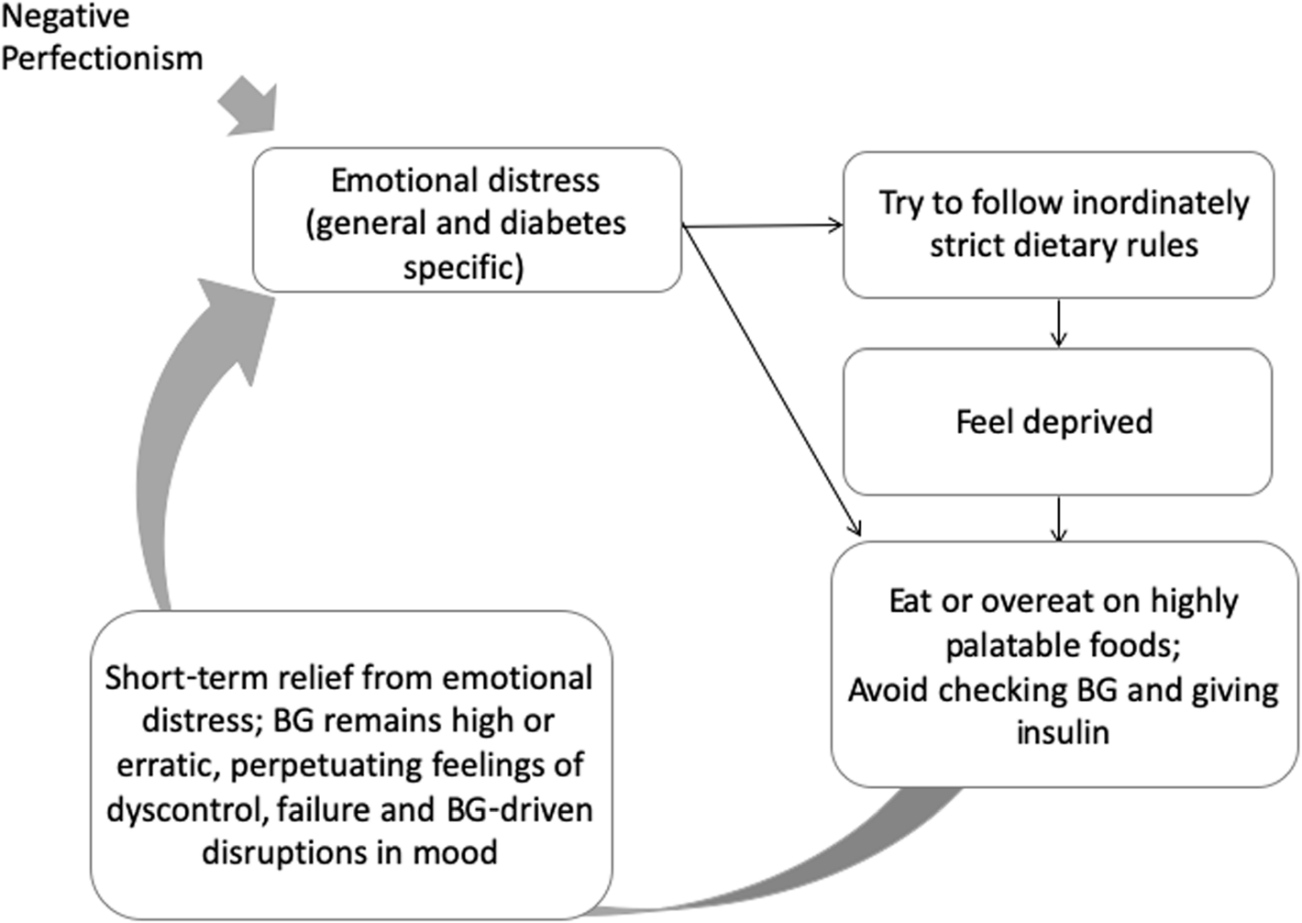
Hypothesized functional pathways of eating disorder behavior in type 1 diabetes

Perfectionism is a trait feature that is highly correlated with EDs in nondiabetic populations [18]. Research differentiates negative perfectionism (i.e., perfectionistic concerns, including fear of failure and making mistakes) and positive perfectionism (i.e., perfectionistic strivings), with a stronger correlation between negative perfectionism and EDs [19]. In the context of T1D, negative perfectionism might lead to extreme responses to the T1D treatment regimen and associated distress that inadvertently contribute to an ED. For example, T1D patients with elevated perfectionism may be more prone to “clamp down” on eating, attempting to follow extremely strict dietary rules (which paradoxically increases risk for bouts of disinhibited eating [20]). Alternatively, they may “give up,” eating or overeating highly palatable foods, and avoid giving insulin to reduce the impact of eating on weight. While these responses may help individuals feel better in the short-term, they would perpetuate poor glycemic control, increasing feelings of failure and further disrupting mood. See Fig. 1.

### A novel treatment approach

Acceptance and Commitment Therapy (ACT) is a contemporary cognitive behavioral therapy that may be well-suited for chronic illnesses such as T1D [21]. While traditional cognitive behavioral therapy focuses on challenging and changing beliefs [22], ACT emphasizes changing how individuals respond to their distressing thoughts/feelings [21]. Thus, ACT may be well-matched to T1D, in which distressing cognitions (e.g., the possibility of medical complications) are often not irrational or illogical and thus not easily disputed, and may be cued multiple times a day as individuals engage in T1D management.

ACT uses acceptance and mindfulness strategies to decouple the typical relationship between distressing thoughts/feelings and behavior, such that behavior may be more flexible and aligned with personal values (i.e., who the person wants to be or how they want to live their lives) [21, 23]. ACT’s values orientation might help T1D patients persist in diabetes management tasks, even when doing so is not immediately reinforcing or is even punishing (e.g., patients may experience edema as they begin to administer more insulin which creates physical and emotional discomfort). The ability to be fully in the present moment with one’s internal experience and choose behavior based on personal values is described as psychological flexibility, and the primary process of change in ACT interventions [21, 23].

Preliminary evidence suggests ACT may improve diabetes management in individuals with type 2 diabetes and that it may be useful for EDs (e.g., [24, 25]), including more intractable conditions such as anorexia nervosa [26, 27]. However, ACT has not been adapted for use with T1D patients with EDs.

### iACT protocol

*i*ACT is a novel treatment protocol based on ACT that was designed to target functional pathways to ED development and maintenance among individuals with T1D. *i*ACT combined individual in-person sessions with a mobile application (“app”) (denoted with the “*i*” in *i*ACT), used in between sessions to facilitate ACT skills practice and behavior change. *i*ACT focused on the unique emotional antecedents and consequences of ED behaviors among individuals with T1D (e.g., feelings of failure in diabetes). Individuals were taught to recognize ED antecedents in the moment and cope more effectively using acceptance and mindfulness skills. The individual’s personal values were also clarified and used to encourage individuals to engage in T1D management tasks, even when it was upsetting (e.g., testing BG or giving insulin after overeating). Acceptance interventions extended to the individual, and encouraged a more compassionate and less punitive approach to managing oneself and T1D (e.g., viewing blood glucose as data rather than a marker of personal success/failure).

The *i*ACT app was a modified version of Recovery Record, an existing mobile app designed to help individuals with EDs recover [28]. The decision to include an app was based on previous research that has found individuals with T1D and ED do not fare well in outpatient treatment, but may do better in inpatient or residential settings (e.g., [29, 30]). These settings have the advantage of providing therapeutic support in the moment that individuals are making decisions about their T1D management. A mobile app can similarly provide additional therapeutic support anytime, including in the late afternoon or overnight when individuals may be more prone to skip insulin [31]. The app was also included to expedite skill acquisition and generalization to daily life (a common obstacle in cognitive behavioral therapy) [32] and it allowed T1D patients to track daily behavior change goals, rather than relying on distal outcomes, such as HbA_1c_. As individuals with T1D engage in new behaviors more regularly, and glycemic control improves, changes may be maintained without app support.

The original Recovery Record app has interactive features such as logs to track thoughts, feelings and behaviors (particularly at mealtimes), the ability to set personal goals, coping skills and affirmations, among other features [28]. This app was modified for our model of ED maintenance in T1D and our strategy for intervention [33]. This included adding T1D-specific feelings (e.g., feeling overwhelmed or like a failure in T1D) to the logs and T1D-specific management behaviors and goals (e.g., taking insulin at each meal). It also included: 1) creating ACT specific coping skills presented at times of heightened emotional distress (e.g., mindfulness exercises, such as watching thoughts like “Leaves on a Stream” [21, 34] or observing emotions as waves) [34], 2) adapting general affirmations to recontextualize T1D with messages of strength, humor or compassion (“*You don’t know how strong you are until strong is the only option you have: TYPE ONE*” [33], and 3) building a new values feature that presented participants’ personalized values images to encourage skill use at times of heightened emotional distress. Before conducting the open trial, the app modifications were vetted with adults with T1D in two focus groups (total *N* = 10) and online questionnaires (*N* = 33). The fully functioning app was then beta tested with 9 T1D patients with eating and weight concerns. Participants used the fully functioning app for 14 days. On average, participants accessed the app 47.56 times over 14 days (or an average of 3.40 times per day) and reported no functionality errors or concerns with the app, indicating it was appropriate to continue to the open trial examining the acceptability and feasibility of the full intervention.

### Study aims

The primary aim of the current study was to examine the acceptability and feasibility of *i*ACT, an app-assisted, tailored ACT intervention for EDs in T1D. Secondary aims of the study were to 1) assess whether the intervention functioned as intended, increasing psychological flexibility with distressing thoughts and feelings about diabetes and decreasing the extent to which these experiences interfered with taking effective action or pursuing values, and 2) the potential of *i*ACT to decrease ED symptoms and improve diabetes management, which has not been shown with previous outpatient treatments. This is the first study of a tailored intervention for EDs in individuals with T1D, which might be more effective in reducing symptoms and improving glycemic control than more generic ED treatments.

## Method

### Participants

Participants were older adolescents and adults with T1D with clinically significant ED symptomatology. To be eligible for the study, T1D patients had to be at least 17 years old, meet diagnostic criteria for an ED, and have a physician managing their diabetes whom they agreed to follow up with throughout the study. They also had to consent to the study team contacting their physician as needed for the study or to maintain safety. Original study criteria also included baseline HbA_1c_ ≥ 7.5% (indicating that average blood glucose over the past 2–3 months was elevated above recommended levels); however, this requirement was dropped because some individuals who presented for the study met full criteria for an ED but did not have HbA_1c_ ≥ 7.5% (due to maladaptive compensatory behaviors, such as severe food restriction between binge episodes that resulted in an overall lower average blood sugar). These individuals were part of the target population and thus included in the study. Individuals with T1D were excluded if they reported an inability to manage their diabetes independently (e.g., due to cognitive impairments) or a thought disorder (e.g., psychosis), or hypoglycemic unawareness (assessed via the Gold Method [35]) which would pose additional safety concerns or suggest another treatment may be more appropriate (e.g., blood glucose awareness training).

The recruitment target was *N* = 25, for a final sample of *N* = 21 with attrition. Assuming two-tailed α = .05, a pre-post mean of average 3-day blood glucose correlation of 0.7, and *SD* = 58.08 mg/dL (calculated from prior data [17, 31], a sample size of 21 provided 80% power to detect a reduction of at least 32 mg/dL in average daily blood glucose, equivalent to a .5% reduction in HbA_1c_, which is considered clinically significant [36].

Participants were recruited from endocrinology clinics at two major medical centers in the Southeast, directly by their treatment providers or by flyers placed in the clinic, or via an email announcement sent to a T1D participant research registry. The study was described as a treatment study for patients with T1D who sometimes take less insulin than they need and/or have concerns about their eating, weight and diabetes management. Interested individuals contacted the Clinical Research Coordinator for eligibility screening. Individuals with Diabetes Eating Problems Survey – Revised (DEPS-R [37];) scores ≥20 were invited onsite for a diagnostic interview to confirm ED diagnosis. Participants provided informed consent for screening and again for study participation. All study procedures were approved by the Institutional Review Board (IRB).

### Eating disorder diagnosis

The Eating Disorder Examination (EDE [38];) is a gold standard semi-structured diagnostic interview that was used to confirm ED diagnosis. The EDE was administered by a Masters or PhD level assessor who was trained in the EDE, and specifically how to administer and score the EDE with T1D patients (e.g., how to discriminate when the individual’s attitudes or behavior are beyond that expected or necessary for diabetes management). Intentionally transdiagnostic, clinical presentation could vary and eligible individuals could present with one or several ED behaviors (e.g., severe caloric restriction, binge eating, self-induced vomiting or insulin restriction for weight control) in any combination if these behaviors were associated with significant distress or impairment.

### Procedure

Individuals who enrolled in the study had height/weight measurements taken and blood drawn to determine HbA_1c_. They also completed questionnaires via Qualtrics® (See Measurements). Participants were then assigned to a therapist (RM, AM). They completed a clinical intake and 12 weekly therapy sessions (50–60 min in duration) with 3 optional tapering sessions (occurring every 2–3 weeks), and used a tailored mobile app between sessions. During the study, participants maintained care with the physician currently managing their diabetes. Medical oversight for study tasks was provided by the study’s endocrinologist (MF) and safety was monitored by an independent safety officer (DS).

Baseline assessments (i.e., EDE, height/weight measurements, questionnaires and HbA_1c)_ were repeated after Session 12 (3 months). This was considered end-of-treatment and the primary assessment point. Assessments (with the exception of the EDE) were repeated at two additional time points, administered 3 months after the previous assessment (approximately 6 and 9 months after treatment initiation). In some cases, HbA_1c_ and height/weight measurements for these assessments were obtained from proximal medical visits (rather than asking participants to repeat these measures within a short time frame).

Participants also completed a questionnaire at each therapy session. The questionnaire asked about frequency and severity of hypoglycemia, edema, and other physical and emotional symptoms and medical utilization (routine and emergency visits). This questionnaire was expanded to include the Valuing Questionnaire (VQ [39];) after participants completed session material reviewing core content (typically Session 2).

### Process of change measures

#### Acceptance and action diabetes questionnaire (AADQ; [23])

The AADQ is a diabetes-specific version of the Acceptance and Action Questionnaire [40], a measure widely used in ACT clinical trials to capture change in psychological flexibility. The AADQ consists of 11 items that measure willingness to experience diabetes-related thoughts and feelings, or the inverse, the extent to which experiential *unwillingness* (rejection or suppression of diabetes-related thoughts and feelings) interferes with diabetes management using a Likert-type scale. Sample items include “When I have an upsetting feeling or thought about my diabetes, I try to get rid of that feeling or thought” and “I do not take care of my diabetes because it reminds me that I have diabetes.” Gregg et al. [24] reported a Cronbach’s α = .94 for the AADQ.

#### Valuing questionnaire (VQ; [38])

The VQ is a 10-item measure that was designed to be administered weekly in clinical and research contexts to capture ongoing change. The VQ measures the extent to which personal values were obstructed by unwanted thoughts and feelings and progress in valued life domains. Unlike the AADQ, the VQ does not require individuals to have explicit awareness of the avoidant function of their behavior, and items extend beyond chronic illness management. Sample items include “I had unpleasant thoughts and emotions that stopped me from achieving my goals.” (Obstruction) and “I made progress in areas of life that I care most about.” (Progress).

### Outcome measures

#### Eating disorder examination (EDE; [37])-global score

In addition to diagnostic information, the EDE quantifies ED symptom severity with 62 items that assess the frequency and severity of disordered eating behaviors and attitudes within the preceding 4 weeks. The EDE has demonstrated high inter-rater reliability and test-retest reliability as well as convergent validity with daily food records and measures of similar constructs [41, 42].

#### Diabetes eating problems survey – revised (DEPS-R; [36])

The DEPS-R is a 16-item self-report measure specifically designed to assess disordered eating in individuals with T1D. Respondents rate the frequency with which they have experienced relevant attitudes and behaviors over the past 4 weeks on a 6-point Likert scale ranging from ‘never’ to ‘always.’ Sample items include: “I feel fat when I take all my insulin,” “I would rather be thin than have good control of my diabetes,” “After I overeat, I don’t take enough insulin to cover my food,” and “I avoid checking my blood sugar when I feel like it is out of range.” Scores range between 0 and 80, with higher total scores indicating more ED symptoms. Scores ≥ 20 are considered clinically significant and have been associated with higher HbA_1c_ [37]. Although originally validated with adolescent patients, the DEPS-R has been used with adults with T1D and has demonstrated satisfactory internal consistency and construct validity among adult samples (e.g., [43]).

#### Diabetes self-management questionnaire (DSMQ; [43])

The DSMQ is a measure of engagement in self-care activities associated with glycemic control. The self-report questionnaire includes 16 items describing self-care behaviors. Respondents rate the extent to which each item applies to them over the past 8 weeks using a four-point Likert scale (3 – ‘applies to me very much’ to 0 ‘does not apply to me’). The DSMQ yields a global score estimate of self-care and five subscale scores (Physical Activity, Dietary Control, Medication Adherence, Physician Contact, and Glucose Management). Schmitt et al. [44] reported good internal consistency (α = 0.84) for the DSMQ.

#### Hemoglobin A1c (HbA_1c_)

Blood for HbA_1c_ assessment was drawn by a trained phlebotomist in the Behavioral Diabetes Research Laboratory and analyzed by LabCorp using the Roche Tina Quant assay.

#### Diabetes distress scale (DDS; [44])

The DDS is a 17-item questionnaire that assesses diabetes-related emotional distress. Sample items include “Feeling angry, scared, and/or depressed when I think about living with diabetes,” and “Feeling that I am often failing with my diabetes regimen.” Respondents rate the degree to which each item was problematic during the last month on a scale ranging from 1 (‘no problem’) to 6 (‘serious problem’). Item scores are averaged resulting in an estimate of total diabetes distress ranging between 1 and 6. Scores of 3 or higher are considered to reflect clinically meaningful levels of distress [45]. Polonsky et al. [46] report good internal reliability and validity (α = 0.93).

#### Patient-reported outcomes measurement information system (PROMIS; [46]) anxiety and depression scales

The PROMIS research tools were developed by the National Institutes of Health (NIH) to allow for comparison of physical, mental, and social health related outcomes across studies. The PROMIS Short Form-Anxiety 8a (PROMIS Anxiety) and PROMIS Short Form-Depression 8a (PROMIS Depression) are self-report instruments, each containing 8 items, which are rated on a 5-point response scale. The Anxiety 8a measures symptoms of anxiety, including fear, nervousness, and tension, whereas the Depression 8a measures symptoms of depression, including worthlessness, helplessness, and unhappiness. Measures assess frequency of symptoms during the past 7 days with response options ranging from 1 (‘never’) to 5 (‘always’). Raw scores of these instruments are converted into standardized *T*-scores, based on a normative sample with a mean of 50 and standard deviation of 10 (higher scores reflect more severe symptomatology). These measures demonstrate robust psychometric properties established by the PROMIS instrument development and validation standards [47].

### Data analysis

Continuous data were examined for normality assumptions and were acceptable with the exception of HbA_1c_, which was transformed (natural log transformation). Paired sample *t*-tests were used to compare participants’ AAQ-D, EDE, DEPS-R, DSMQ, HbA_1c_, DDS, and PROMIS scores at baseline and end-of-treatment (Session 12, 3 months) and Cohen’s *d* effect sizes were calculated. In the case of an outlier, analyses were run with and without the outlier (which did not change the results). Data from the VQ were first analyzed using a flexible curve fitting procedure (3 knot restricted cubic spline) to determine whether a linear model was adequate. Data from the VQ were then analyzed using mixed models. Session number was treated as a continuous variable, with the intercept and session specified as random effects. An unstructured covariance matrix was used based on a comparison of fit indices.

Data from one participant who completed treatment was excluded from all analyses due to late reveal of psychosis which made her ineligible for the study. This individual had paranoid delusions directly related to her insulin pump which interfered with her diabetes management.

## Results

### Sample characteristics

Complete demographics are provided for the full recruited sample (*n* = 28) as well as treatment completers (*n* = 20) for comparison purposes (Table 1). The recruited sample was 79% White and 100% female. Average age of onset of T1D was 16.2 (*SD* = 11.6). Thirty-nine percent of the sample (*n* = 11) reported using an insulin pump as their mode of insulin delivery, rather than multiple daily injections, which has been associated with lower HbA1_c_ [48]. Seventy-five percent (*n* = 21) reported that they had major or minor complications associated with diabetes; 7.1% (*n* = 2) indicated they did not have any diabetes-related complication; 17.9 (*n* = 5) did not complete this part of the assessment. The most common complications were diabetes ketoacidosis after initiation of insulin therapy (diabetic ketoacidosis prior to diagnosis is not included here) (50%), bladder, yeast or other urinary tract infection (46%), stomach or intestinal problems (39%) and retinopathy (36%). Skin problems or infections, slow healing cuts or other wounds and decreased vision were also common, with 25% of the sample reporting each of these complications. A few participants also reported neuropathy (11%) and nephropathy (7%).

**Table 1:** *Participant Demographics*

	*All Recruited Participants* (*n* = 28)*	*Treatment Completers* (*n =* 20)	*Analyzed* (*n* = 19)
Characteristic	*M* (*SD*) or %	*M* (*SD*) or %	*M* (*SD*) or %
Age (yrs.)	34.8 (12.4)	35.0 (11.8)	35.4 (12.0)
Sex (% female)	100	100	100
Race/Ethnicity (%)			
Caucasian/White	78.6	80	78.9
African-American/Black	14.3	10	10.5
More than one race	7.1	10	10.5
Marital Status (%)			
Never Married	46.4	50	47.4
Married	25.0	35	36.8
Separated/Divorced	14.3	15	15.8
Unknown	14.3	0	0
Highest Level of Education (%)			
Some high school	3.6	5	5.3
Some college/technical school	25.0	20	21.1
Bachelor’s degree	28.6	35	31.6
Graduate degree	28.6	40	42.1
Unknown	14.3	0	0
Body Mass Index (BMI) (*M*)	27.8 (5.5)	28.2 (5.8)	28.5 (5.7)
Normal Weight (%)	29	35	32
Overweight (%)	25	25	26
Obese (%)	29	40	42
Unknown (%)	18	0	0
Insulin Administration (%)			
Insulin Pump	39	45	47
Multiple Daily Injections	46	55	53
Unknown	14	0	0
Age at Type 1 Diabetes Diagnosis (Years)	16.2 (11.6)	17.2 (12.2)	17.3 (12.5)
Diabetes Complications (%)			
History of Retinopathy	36	40	42
History of Neuropathy	11	10	11
History of Nephropathy	7	5	5
History of Diabetes Ketoacidosis Post Initiation of Insulin Therapy	50	55	58
Eating Disorder Diagnosis (%)			
Bulimia Nervosa	46	60	63
Subthreshold Bulimia Nervosa	18	15	11
Purging Disorder	11	15	16
Binge Eating Disorder	7	10	11
Unknown	14	0	0

*Note:* Data analyses were conducted on 19 of the 20 treatment completers; *n* = 1 was excluded from analyses due to late reveal of psychosis directly impacting her management behavior

Participants reported clinically significant ED symptomatology with DEPS-R scores ranging from 25 to 76 (*M* = 41.25; *SD* = 12.84). Of the 24 participants who completed the diagnostic interview, the majority (75%) met criteria for threshold (*n* = 13) or subthreshold (*n* = 5) bulimia nervosa. Subthreshold bulimia was defined as meeting all criteria for bulimia but with binge-purge frequency less than what is required for full diagnosis. Thirteen percent (*n* = 3) met criteria for purging disorder, 8% (*n* = 2) met criteria for binge eating disorder, and 4% (*n* = 1) did not meet threshold criteria for an ED (this participant was withdrawn from the study by the PI and referred to more appropriate treatment). Twenty-five percent of the full sample (*n* = 28) reported receiving treatment for an ED in the past (18%, *n* = 5 did not provide this information).

HbA_1c_ for the sample ranged from 6.1–15.5% (*M* = 9.93, *SD* = 2.81). As previously reported, five participants had HbA_1c_ below 7.5%. These individuals were either restrictive in their eating, or vacillated between binge eating and maladaptive compensatory strategies of self-induced vomiting, overexercise or fasting, which resulted in significant daily BG variability, but produced an average BG in an acceptable range. ED symptoms in the absence of elevated HbA_1c_ is perhaps the most challenging clinical presentation because problems may go undetected by physicians if patients are not regularly screened for ED symptoms.

The sample was highly distressed about their diabetes, with baseline scores on the DDS ranging between 2.12 and 4.88, with the average falling above 3, the clinically recommended cut-off for “high diabetes distress” (*M* = 3.79, *SD* = .74) [45]. Baseline descriptives for all measures at major assessment points are provided in Table 2.

**Table 2:** Mean (SD) for Outcome Variables for the Recruited and Analyzed Sample at all Assessment Points

	Baseline (*n* = 28)*	Baseline (*n* = 19)	End-of-Treatment (3 Months) (*n* = 19)	Second Assessment (6 Months) (*n* = 16)	Third Assessment (9 Months) (*n* = 15)
EDE	3.5 (1.0)	3.5 (1.1)	2.5 (1.0)	-	-
DEPS-R	41.3 (12.8)	42.7 (13.9)	25.3 (10.8)	26.3 (11.8)	28.3 (14.9)
DSMQ	4.7 (1.6)	4.7 (1.5)	6.3 (1.5)	6.1 (1.8)	6.4 (1.0)
HbA_1c_ (%)	9.9 (2.8)	9.8 (2.7)	8.9 (2.4)	8.5 (1.9)	9.3 (2.4)
DDS	3.8 (.7)	3.8 (.6)	2.8 (.7)	2.7 (.7)	2.7 (.93)
PROMIS-D	60.2 (6.7)	61.0 (6.9)	57.0 (7.7)	56.5 (8.2)	55.8 (11.3)
PROMIS-A	61.2 (7.3)	60.84 (7.7)	58.5 (10.2)	56.1 (8.5)	58.5 (10.4)
AADQ	49.9 (7.7)	49.2 (6.9)	56.1 (4.9)	56.1 (6.1)	56.2 (7.5)

Note: *Includes all recruited participants who completed some or all of the baseline assessment

*EDE* Eating Disorder Examination Global Score (administered only at baseline and EOT), *DEPS-R* Diabetes Eating Problems Survey-Revised, *DSMQ* Diabetes Self-Management Questionnaire (higher scores indicate improvement), *HbA*_*1c*_ Hemoglobin A_1c_, *DDS* Diabetes Distress Scale, *PROMIS-D* PROMIS Short Form-Depression 8a, *PROMIS-A* PROMIS Short Form-Anxiety 8a, *AADQ* Acceptance and Action Diabetes Questionnaire (higher scores indicate improvement)

### Recruitment and retention

Five of the 28 individuals recruited for the study withdrew after completing some or all of the baseline assessment but before starting treatment, for personal reasons unrelated to the intervention (e.g., distance to travel). Of the 23 individuals who started treatment, 87% *(n* = 20) completed treatment. As previously noted, one participant (4%) was withdrawn by the PI at mid-treatment and referred for more appropriate treatment (this participant had attention-deficit disorder as the primary issue impacting T1D management and was determined to not meet criteria for an ED), and two participants (9%) withdrew after beginning treatment (one withdrew after completing the first session and the other was lost to contact after completing two sessions).

The majority of treatment completers (*n* = 18) completed optional tapering sessions, and over half of these individuals (*n* = 12; 60%) requested to continue treatment beyond this point. Given the lack of information about appropriate treatment duration for T1D patients with EDs, the risk of continued ED symptoms (e.g., insulin restriction), and the lack of other treatment options for this high-risk patient population, participants who requested to continue to receive treatment were provided with additional therapy sessions. The total number of sessions among treatment completers (*n* = 20) ranged from 12 to 21, with the exception of two participants who continued in weekly treatment after their end-of-treatment (Session 12, 3 month) assessment. Both of these individuals presented with additional comorbid diagnoses (e.g., major depressive disorder) and complex trauma.

### App engagement

All participants who initiated treatment (*n* = 23) downloaded and accessed the app at least once, with the exception of the one participant who discontinued treatment after two sessions. There was considerable variability in app use as indexed by completion of logs providing information about meals, ED and diabetes management behaviors or emotional distress. Treatment completers (*n* = 20) completed 156.2 (*SD* = 109.35; Median = 161.50) logs on average between Session 1 and Session 12, and averaged 189.9 (*SD* = 152.86; Median = 196.5) logs between Session 1 and the last study assessment (occurring approximately 9 months after treatment initiation).

### Treatment tolerance

Overall, treatment was well tolerated. Therapy session questionnaires indicated mild to moderate (but not severe) bloating and edema were common, particularly mid-treatment. Hypoglycemia was also not a major problem. Six participants reported hypoglycemia was a regular occurrence (occurring multiple times a week). About half the sample (*n* = 10) reported hypoglycemic events were absent or infrequent throughout treatment. Four participants reported a mixed pattern (i.e., hypoglycemic events were absent or infrequent about 40–60% of the time, and occurred multiple times a week the remainder of the time). Participants did not require emergency intervention for hypoglycemia during the study, but one participant did have a severe episode that required this type of intervention during the follow-up period.

No participants were hospitalized during the intervention. One participant (a 17-year old with bulimia nervosa) sought residential treatment after Session 12. This participant improved her HbA_1c_ during the 12 weeks, and entering residential treatment was viewed as a positive next step to continue her progress.

BMI did not significantly change from baseline to end-of-treatment, *t* [19] = − 1.3, 95% CI [−.96, .25], *p* = .23, which is important given weight gain is often a concern for ED patients that may interfere with treatment engagement.

### Completion of assessments

Of the participants who completed treatment (*n* = 20), 100% completed the first assessment (Session 12, 3 months). Ninety percent (*n* = 18) completed some or all of the second assessment (a few participants provided a blood sample for HbA_1c_ but did not complete the questionnaires or vice versa), scheduled 3 months after the individual’s previous assessment. Eighty percent (*n* = 16) completed some or all of the third assessment battery, scheduled 3 months after the individual’s previous assessment. Participants who did not complete all study assessments were unable to do so for the following reasons: family member illness and associated time restrictions (*n* = 1), relocation for residential treatment (*n* = 1) and unknown reasons (i.e., lost to contact) (*n* = 2).

### Changes in psychological flexibility

Participants reported increased psychological flexibility with diabetes-related thoughts and feelings from baseline to end-of-treatment, as indexed by the AADQ, *t* [19] = − 4.1, 95% CI [− 10.53, − 3.37], *p* = .001, Cohen’s *d* = 0.94.

Preliminary analyses of the VQ suggested a linear fit was probably adequate (i.e., the trajectory of change for either VQ subscale did not appear to be nonlinear, Obstruction *p* = 0.89 and Progress, *p* = 0.28). The linear slope coefficient for Obstruction was − 0.51 (95% CI = − 0.79, − 0.24, *p* = 0.001), indicating that Obstruction of Values was reduced by about a half point per session. For Progress, the linear slope coefficient was 0.30 (95% CI = 0.09, 0.51, *p* = 0.006), indicating that Progress in Values increased by about a third of a point every session.

### Eating disorder symptoms

Participants reported significant improvements in ED symptoms from baseline to end-of-treatment, as indexed by the EDE Global Scores, *t* [19] = 4.13, 95% CI [.51, 1.57], *p* = .001, Cohen’s *d* = 0.90, and the DEPS-R, *t* [19] = 7.8, 95% CI [12.73, 22.11], *p* < .001, Cohen’s *d* = 1.79.

### Diabetes management

Diabetes self-management (DSMQ scores) significantly improved, *t* [19] = − 4.1, 95% CI [− 2.36, −.76], *p* = .001, Cohen’s *d* = 0.92. Change in HbA_1c_ did not reach statistical significance, *t* [19] = 1.9, *p* = .08, Cohen’s *d* = 0.44. Mean decrease in HbA_1c_ was .9% (95% CI = -.07–1.77). Fifty-three percent of the participants with HbA_1c_ ≥ 7.5% at baseline evidenced at least a .5% decrease from baseline to end-of-treatment, which is considered clinically significant [25].

### Emotional distress

Diabetes distress (DDS scores) decreased from baseline to end-of-treatment, *t* [19] = 6.0, 95% CI [.69, 1.45], *p* < .001, Cohen’s *d* = 1.38. There were mixed findings for change in depression and anxiety, which were not explicitly targeted by the intervention. Depression (PROMIS Depression scores) significantly improved, *t* [19] = 2.4, 95% CI [.51, 7.32], *p* = .03, Cohen’s *d* = 0.55, but anxiety (PROMIS Anxiety scores) did not, *t* [18] = 1.0, 95% CI [− 2.4, 7.08], *p* = .32, Cohen’s *d* = 0.24).

## Discussion

The problem of EDs in T1D is significant and relatively intractable to change [4–6]. Treatments for this high-risk patient population are underdeveloped and understudied, and thus patients are without empirically-based outpatient treatment options. The current study piloted a novel intervention tailored to the unique experience of living with diabetes and the related emotional distress that may contribute to the development of maladaptive eating and weight control behaviors. This is the first study to assess the acceptability and feasibility of an outpatient intervention specifically tailored to this unique patient population. The key finding was that treatment was acceptable to T1D patients with ED symptoms and feasible to implement with minimal complications.

Changes in target processes of psychological flexibility and value-guided action were also observed, and individuals who participated in the intervention experienced significant improvements in ED symptomatology, diabetes management and diabetes distress. However, given the open trial design, we cannot infer that changes were the result of intervention, rather than nonspecific treatment effects, natural course, or regression to the mean; although it should be noted that previous studies have found that without intervention, ED behaviors in T1D tend to persist and worsen over time [4].

Many participants requested additional treatment. This suggests that the treatment was acceptable, but also indicates that participants believed that they needed more or continued support. Previous reviews have indicated typical treatment duration of 8 to 20 sessions for EDs, depending on clinical presentation [49–52]. Thus, in comparison, *i*ACT might be considered brief. Longer treatment duration could be helpful for some patients.

The current treatment paired individual therapy sessions with the use of a mobile app between sessions to evoke and reinforce behavior change in the natural environment. It did not test the individual components of the intervention, and thus it is unclear whether particular components are necessary or sufficient. This would be an important next step for potentiating treatment effects and maximizing treatment efficiency. Future studies might examine whether the app is necessary, whether engagement with particular app features predicts outcomes, or explore strategies to increase app use among some participants. Future studies might also explore whether some individuals respond to the app alone, or whether the app can be paired with a single in-person contact or with video/telehealth sessions to enhance portability. Future studies could examine the effect of the different treatment components, as well as treatment dose, using adaptive (e.g., Sequential Multiple Assignment Randomized Trial; SMART) designs.

The primary aim of the current study was to assess acceptability and feasibility of this treatment approach. However, the outcomes for HbA_1c_ are still of interest. Many participants evidenced change of .5% in HbA_1c_ from baseline to end-of-treatment, which is considered clinically significant. However, mean change in HbA_1c_ did not achieve statistical significance. The more modest effect size for HbA_1c_ (relative to the large effects observed for other outcomes) may be attributable to the amount of time necessary for behavior change to be reflected in HbA_1c_ (with the average lifespan of a red blood cell 100–120 days). Longer follow up in controlled studies is needed.

Change in HbA_1c_ might have also been attenuated due to the inclusion of patients with HbA_1c_ < 7.5%. These patients were part of the population of interest (i.e., they met criteria for an ED) and their clinical presentation put them at risk for complications associated with both hyper- and hypoglycemia due to significant BG variability associated binge eating, purging or other ED behaviors. Future studies of EDs in T1D should adequately account for these patients in power calculations and use continuous glucose monitoring, in addition to HbA_1c_, to index treatment related improvements in glycemic control. For example, continuous glucose monitoring might reveal decreased time spent below 70 or above 180 mg/dL, or reductions in daily BG variability as a result of intervention. This is increasingly possible with technological advancements that allow glucose sensors to be worn for longer periods of time and data loss to be minimized.

The current study allowed participants to continue in treatment if they requested to do so. This decision was made prioritizing the needs of this high-risk population, and with the understanding that necessary and appropriate treatment duration for T1D patients with EDs is unknown and may vary by individual. As with any pilot study, findings should be interpreted with caution.

The current study may have limited generalizability as the majority of participants were white females. While this is expected given that T1D is more prevalent in white individuals, and EDs are more prevalent among women and girls [53, 54], larger studies with more diverse samples are needed. The current study also recruited participants from endocrinology clinics. Individuals recruited from endocrinology may be less severe than those who would be recruited from a general psychiatry or ED practice, or have other differences that limit the generalizability of the findings.

As first noted, the most significant limitation to the current study is the single-arm trial design. However, the primary aim of the investigation was to determine whether this type of treatment is acceptable and feasible, and gather preliminary evidence that the targeted processes of change were engaged and that treatment might improve ED symptoms and T1D management. Findings from the current study suggest that a randomized controlled trial of *i*ACT is warranted.

## Conclusion

In summary, *i*ACT holds promise for the treatment of ED symptoms among T1D patients. This treatment approach, which is tailored to this unique patient population and grounded in theory regarding how these behavior problems may develop or be maintained, is acceptable to T1D patients and feasible to implement. Additional research is needed to test whether improvements in ED symptoms and glycemic control exceed what would be expected with time alone or with another treatment. Future research might also investigate which treatment components are necessary and sufficient, explore options for maximizing treatment effects or portability of the intervention, and examine patient matching to treatment and treatment dose. More broadly, this study adds to a growing body of research on ACT for a variety of clinical problems [23], including EDs and diabetes management. It also provides an example of the use of mobile technology in ACT-based treatment delivery, which may inform innovative, highly scalable interventions.

## Data Availability

The dataset used and analyzed in the current study is available from the corresponding author upon request.

## Abbreviations

T1D: Type 1 diabetes
EDs: Eating disorders
ED: Eating disorder
BG: Blood glucose
HbA_1c_: Hemoglobin A1_c_
App: Mobile application
